# Supplementary material for: Resveratrol Alleviates the Early Challenges of Implant-Based Drug Delivery in a Human Glial Cell Model
Source: Int J Mol Sci. 2024 Feb 8;25(4):2078. doi: 10.3390/ijms25042078 (PMC10889494; doi:10.3390/ijms25042078)

Figure S1: Secondary antibody controls for immunocytochemistry shown in Figures 3 and 5. The primary antibodies were omitted during the staining procedure. Representative images from n = 3 independent cell cultures (scale bar: 50 µm)

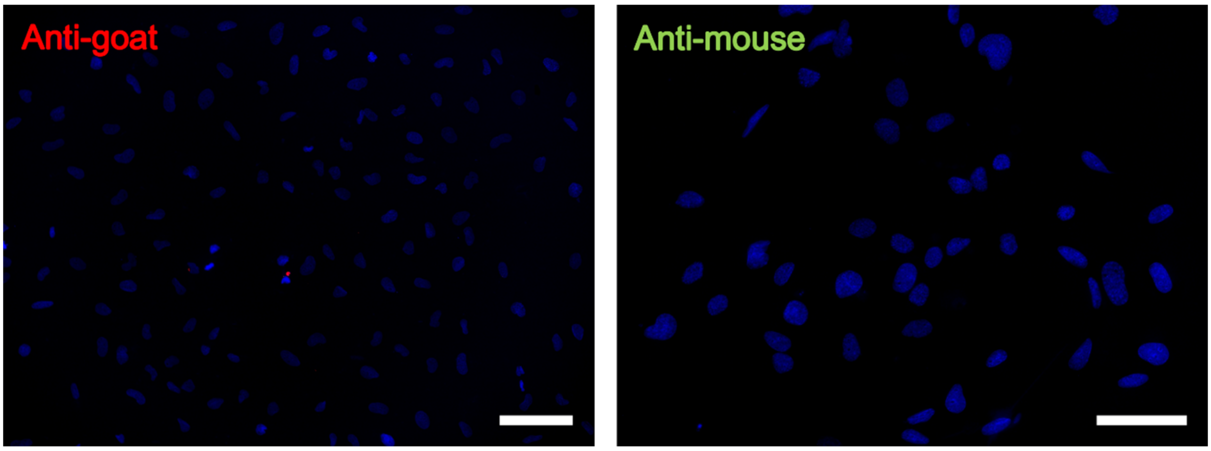

Supplement: Supplementary file 1 [file ijms-25-02078-s001.zip › Figure S1.pdf]
