# Supplementary material for: Resveratrol Alleviates the Early Challenges of Implant-Based Drug Delivery in a Human Glial Cell Model
Source: Int J Mol Sci. 2024 Feb 8;25(4):2078. doi: 10.3390/ijms25042078 (PMC10889494; doi:10.3390/ijms25042078)
Supplement: Supplementary file 1 [file ijms-25-02078-s001.zip › Table S1.pdf]

TableS1: PEG400 does not have a significant effect on HMC3 and SVGA.

HMC3:

Mean +/- SD	Control	+ PEG	$p < 0.05$?	OGD	OGD + PEG	$p < 0.05$?
ROS FACS (MFI)	1843 +/- 161.9	1502	ns	6719 +/- 1284	5648 +/- 537.6	ns
Cytokines qPCR (ΔΔct)	IL1β: 1<br>IL6: 1	IL1β: 1,232 +/- 0.8887<br>IL6: 1.213 +/- 0.6929	IL1β: ns<br>IL6: ns	IL1β: 3,240 +/- 1.159<br>IL6: 2.099 +/- 1.007	IL1β: 2,738 +/- 1.760<br>IL6: 2.105 +/- 1.591	IL1β: ns<br>IL6: ns
Cytokines ELISA (pg/mg)	IL1β: 9,551 +/- 3.258<br>IL6: 68.01 +/- 4.028	IL1β: 9,095 +/- 1.336<br>IL6: 68.51 +/- 10.72	IL1β: ns<br>IL6: ns	IL1β: 85,49 +/- 5.778<br>IL6: 330.4 +/- 38.84	IL1β: 71,23 +/- 20.32<br>IL6: 340.3 +/- 21.05	IL1β: ns<br>IL6: ns
Caspases3/7 activity (RLU)	26703 +/- 5847	26896 +/- 5642	ns	27693 +/- 4394	24060 +/- 4483	ns
Proliferation (n-fold)	14,44 +/- 4,457	11,75 +/- 3,566	ns	6,303 +/- 1,516	5,475 +/- 2,422	ns

HIF-1α ELISA (pg/mg)	537,9 +/- 94,95	560,5 +/- 72,98	ns	453,1 +/- 160,2	333,3 +/- 109,9	ns

SVGA:

Mean +/- SD	Control	+ PEG	p < 0.05?	OGD	OGD + PEG	p < 0.05?
ROS FACS (MFI)	2329 +/- 232.2	2011	ns	8066 +/- 1071	8343 +/- 2587	ns
Galectin-3 qPCR (ΔΔct)	1,000	0,9872 +/- 0.3951	ns	1,727 +/- 0.4713	1,853 +/- 0.4840	ns
Galectin-3 ICC (FI/ count)	163004 +/- 42915	181920 +/- 36675	ns	303700 +/- 51657	231984 +/- 75044	ns
Caspases3/ 7 activity (RLU)	15401 +/- 2217	13403 +/- 1841	ns	33587 +/- 10591	30019 +/- 4497	ns
Proliferation (n-fold)	8,559 +/- 0,9907	11,59 +/- 3,497	ns	6,503 +/- 1,055	7,722 +/- 2,316	ns
HIF-1α ELISA (pg/mg)	406,6 +/- 31.07	383,6 +/- 155	ns	1095 +/- 207.5	734,9 +/- 155.6	ns

HIF-1α ICC (FI/ count)	237881 +/- 119885	166139 +/- 144429	ns	637951 +/- 189280	656002 +/- 211290	ns
